# Catching Nucleosome by Its Decorated Tails Determines Its Functional States

**DOI:** 10.3389/fgene.2022.903923

**Published:** 2022-07-14

**Authors:** Parveen Sehrawat, Rahul Shobhawat, Ashutosh Kumar

**Affiliations:** Department of Biosciences and Bioengineering, Indian Institute of Technology Bombay, Mumbai, India

**Keywords:** nucleosome, PTMs, histone tails, acetylation, acylation, methylation

## Abstract

The fundamental packaging unit of chromatin, i.e., nucleosome, consists of ∼147 bp of DNA wrapped around a histone octamer composed of the core histones, H2A, H2B, H3, and H4, in two copies each. DNA packaged in nucleosomes must be accessible to various machineries, including replication, transcription, and DNA damage repair, implicating the dynamic nature of chromatin even in its compact state. As the tails protrude out of the nucleosome, they are easily accessible to various chromatin-modifying machineries and undergo post-translational modifications (PTMs), thus playing a critical role in epigenetic regulation. PTMs can regulate chromatin states *via* charge modulation on histones, affecting interaction with various chromatin-associated proteins (CAPs) and DNA. With technological advancement, the list of PTMs is ever-growing along with their writers, readers, and erasers, expanding the complexity of an already intricate epigenetic field. In this review, we discuss how some of the specific PTMs on flexible histone tails affect the nucleosomal structure and regulate the accessibility of chromatin from a mechanistic standpoint and provide structural insights into some newly identified PTM–reader interaction.

## 1 Introduction

In the eukaryote’s nucleus, DNA is packaged into the macromolecular “beads on a string”-like structure called chromatin using highly basic histone proteins. A nucleosome is the basic and efficient unit of this organization in which 145–147 bp of DNA are wrapped around a histone octamer (two molecules of each histone H2A, H2B, H3, and H4). Two pairs of H3–H4 dimer form a tetramer stabilized by a characteristic hydrophobic four-helix bundle structure between H3 and H3ʹ, and then two dimers of H2A–H2B interact with H3–H4 tetramer on each side through a second homologous hydrophobic four-helix bundle structure between H2B and H4, forming a globular octamer from which disordered tails protrude out. Through extensive hydrogen-bonding and electrostatic interactions, histones coordinate with DNA *via* conserved histone fold domains, resulting in the bending of negatively charged DNA over a positively charged octamer surface. This bent conformation of DNA brings the phosphate backbone of the two strands closer, and this energetically constrained conformation is maintained by neutralizing negative charges by positively charged lysine and arginine side chains (Luger et al., 1997).

These strong and extensive interactions render the nucleosome a stable disc that can sterically inhibit the binding of chromatin-associated proteins (CAPs). Virtually all eukaryotic organisms use the inhibitory nature of this packaging to regulate access to DNA. However, the information encoded inside the DNA must be retrieved at appropriate times. Although DNA is very tightly compacted, it still remains accessible to many enzyme machineries that replicate it, repair it, and use it to produce RNA molecules and proteins. For doing this, chromatin and nucleosomes must be inherently dynamic and highly malleable. Numerous biochemical and structural studies established the dynamic nature of the nucleosome in terms of its conformation and composition. The disordered N-terminal tail of histones have an affinity to DNA, forming a dynamic complex with DNA termed as “fuzzy conformational ensembles,” which regulate the chromatin structure and dynamics (Ghoneim, Fuchs, and Musselman 2021; Peng et al., 2021; Shukla, Agarwal, and Kumar 2022). Polach and Widom, (1995) demonstrated the phenomenon of intrinsic structural dynamics of nucleosome known as “DNA breathing,” i.e., partially unwrapping and rewrapping of DNA spontaneously. This dynamic unwrapping/rewrapping phenomenon is exploited by several DNA-binding proteins like transcription factors in a tunable and analogous fashion. Using FRET experiments, the Langowski group showed that disassembly of nucleosome is initiated by DNA breathing resulting in a dynamic “octasome,” which opens on a 50 µs time scale at an angle of ≈20°. This results in disruption of dimer tetramer interface with H2A–H2B dimer evicting first followed by H3–H4 tetramer removal (Böhm et al., 2011; Gansen et al., 2018).

In addition to DNA breathing, cells have also evolved various other mechanisms to make nucleosomal DNA more accessible: histones posttranslational modifications, histone chaperones, histone variants, and chromatin remodelers. These regulatory mechanisms control the genome function without changing the nucleotide sequence, also referred to as “epigenetic” marks. Histone post-translational modification is the process of covalently attaching adducts like methyl group, acetyl group, phosphate group, and ubiquitin group. These modifications present on free N-terminal tails or inside the histone fold domain affect the structure and dynamics of nucleosomes locally and chromatin globally and provide the binding platform for different groups of proteins like transcription factors, chromatin remodelers, histone-/DNA-modifying enzymes, and chaperones, especially the charge-altering PTMs inside the globular histone octamer core can modify the electrostatic interaction of histone–histone or histone–DNA, thereby altering the structure and dynamics of nucleosomes (Fenley et al., 2018). For instance, phosphorylation in combination with acetylation inside the nucleosomal DNA entry–exit site modulates DNA accessibility by transcription complexes (Brehove et al., 2015). Misregulation of these modifications can cause many diseases like cancer; therefore, regulating this epigenetic mark is necessary for proper functioning. In this review, we have discussed the role of four PTMs (acetylation, acylation, serotonylation, and methylation) present on flexible and intrinsically disordered histone tails in regulating chromatin accessibility and function. Several excellent reviews on other modifications like phosphorylation (Sawicka and Seiser 2014; Treviño, Wang, and Walker 2015), SUMOylation (Ryu and Hochstrasser 2021), and ubiquitination (Mattiroli and Penengo 2021) are good read to get a better understanding.

## 2 Histone Tails and PTMs

The nucleosome is a globular structure, but the unstructured N-terminal tail of each histone protrudes out from its core. The pioneering work of Vincent Allfrey in the 1960s and subsequent studies revealed that these tails are subjected to many post-translation modifications like acetylation, methylation, and phosphorylation, thus acting as a hub of chromatin signaling (Millán-Zambrano et al., 2022). Covalent modifications of histone tails can alter the chromatin structure *via* cis-effects or trans-effects. Cis-effects are employed by changing the biophysical properties of modified histone chains, like altering the electrostatic charge or structure of the tail, which in turn affects internucleosomal contacts. For example, histone acetylation on lysine residue exerts its effect by neutralizing the positive charge of histone tails. Charge-neutralized tails generate a localized decondensation of the chromatin fiber, resulting in better availability of DNA double helix to the transcription machinery. Acetylation at H4K16 inhibits the packaging of a nucleosomal array in a compact 30-nm chromatin fiber *in vitro* and further abolishes cross-fiber interactions (Michael et al., 2006). In fact, out of four acetylations possible in the H4 tail, K16 acetylation is unique as only this modification reduces the cation-induced folding of the 12-mer nucleosome array implicating cis-effect of acetyl mark (Allahverdi et al., 2011). Multiscale computational studies supported by NMR experiments revealed that acetylated H4 tails lose local contacts and reduced tail availability for forming critical internucleosomal interactions resulting in the unfolding of chromatin fiber (Collepardo-Guevara et al., 2015; Bascom and Schlick 2018). Similarly, phosphorylation adds a net negative charge generating “charge patches,” which result in alteration of nucleosome packaging (Dou and Gorovsky 2000). Bulky groups, such as ADP-ribose and ubiquitin, also affect the arrangements of the histone tails and open up nucleosome arrays.

Histone modifications also act *via* trans-effects, where modification-binding partners are recruited to the chromatin. This is similar to “reading” a specific covalent histone mark by modification reader proteins. For example, the acetylation mark is read by proteins having “bromodomains” (Jacobson et al., 2000). Similarly, methylated lysine or arginine residues are read by chromodomains or similar domains (e.g., MBT and Tudor) to facilitate the modulation of chromatin (Maurer-Stroh et al., 2003). Acetylation, methylation, and phosphorylation were the initially detected and extensively studied modifications. Recent advancement in the high-sensitive mass spectrometry technique has played a pivotal role in revealing a wide array of new modifications, including ubiquitylation, SUMOylation, ADP-ribosylation, a dozen of various acyl groups, serotonylation, and lactylation (Zhao and Garcia 2015). Based on diversity and biological specificity of distinct modifications, Strahl and Allis, (2000) proposed the “histone code” hypothesis, which states that “multiple post-translational modifications form a specific pattern either in combination or sequential fashion on same or different histone tail, to perform a specific downstream function.” The key players involved in defining the histone code are the enzymes or proteins that write, read, and then erase these marks in a specific sequence or modification. This fine-tuned action is critical for regulating most nuclear processes, including replication, recombination, DNA damage and repair, transcription, and differentiation.

The crosstalk and specific recognition of histone PTM by its cognate reader define the temporal and spatial modulation of the genome. After the initial discovery of the bromodomain as an acetylation mark reader and chromodomain as a methylation mark reader, several epigenetic studies have identified a diverse repertoire of “readers” regulating the dynamic nature of the chromatin landscape. For example, RAG2 protein of RAG1/2 V(D)J recombinase reads H3K4me3 modification and induces V(D)J recombination at the T- and B-cell receptor gene locus. Mutations in the reader motif of RAG2 impair V(D)J recombination and can result in immunodeficiency syndromes (Matthews et al., 2007). Many chromatin-associating multi-subunit enzymatic complexes contain a set of multiple readers within one or different subunits, and these readers having specificities for different marks can be in close proximities. These complexes can be “writers” or “erasers” [histone acetyltransferases and histone deacetylases] that can redefine the epigenetic landscape by adding or removing modifications at different sites or chromatin remodelers that can alter the structure and dynamics of chromatin. Combinatorial readout of multivalent histone PTMs on the same tail or at a different tail can provide a lock and key type mechanism to carry out a specific biological function at targeted genomic loci. Owing to their fundamental role, any misreading of these epigenetic modifications has been shown to contribute to many human diseases, including cancer and developmental and autoimmune disorders (Chi, Allis, and Wang 2010; Shen and Laird 2013).

In some cases of acute myeloid leukemia, the reader module of H3K9 trimethylation (PHD motif) is found to be fused with nuclear pore protein (NUP98). This fusion protein remains bound to H3K9me3, interfering with the removal of this modification and the addition of H3K27me3, thereby affecting the normal differentiation of progenitor and hematopoietic cells (Wang et al., 2009). In fact, misinterpretation of acetyl marks by their respective reader domains has been implicated in uterine, bladder, cervical, and other tumors (Zhao et al. 2021). Targeting one of the bromodomain protein families BET (bromodomain and extraterminal domain) by small molecules resulted in the reversal of a cancer cell phenotype in the patient-derived NUT midline carcinoma cell line (Filippakopoulos et al., 2010). Understanding the basic aspects of epigenetic control and the genesis of epimutation-induced human disorders requires an understanding of the molecular mechanism and functional importance of PTM–reader interactions. In the following sections, we discuss the molecular mechanism of PTM readout by different reader modules and the functional significance of these newly identified PTM–reader interactions.

## 3 Acetylation

**FIGURE 1:**
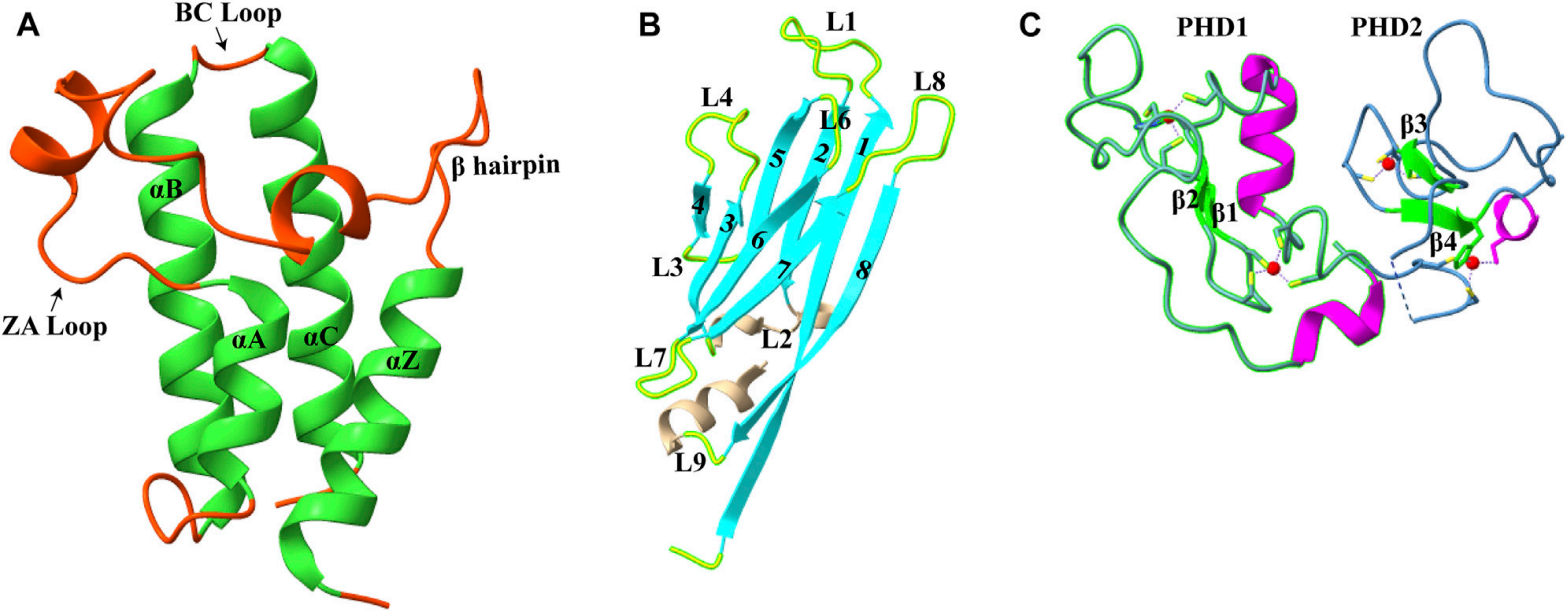
Structures of acetyllysine reader modules **(A)**. Bromodomain of YEATS Sth1 (RSC remodeler): four helices are shown in green (PDB: 6KMB) **(B)**. YEATS domain of AF9 protein (PDB: 4TMP) **(C)**. DPF domain of MOZ protein: antiparallel β-sheets are shown in green, and zinc ions are shown in red color (PDB: 4LJN).

Acetylation of the lysine residue at the ɛ-amino group was the first PTM discovered in thymus histones by Philips in 1961 (Allfrey et al., 1964). The negative charge on the acetyl group neutralizes the positive charge of the lysine side chain, thereby altering the electrostatic properties of histone proteins. This modification is generally correlated with a transcriptionally active state, and the turnover of this modification is controlled by two groups of enzymes: histone acetyltransferases (HATs) and histone deacetylases (HDACs) (discussed in detail in Marmorstein and Zhou, (2014) and Xia et al. (2020)). This mark is found on all histone tails H2A (K5 and K9), H2B (K5, K12, K15, K16, K20, and K120), H3 (K4, K9, K14, K18, K23, K27, K36, and K56), and H4 (K5, K8, K12, K16, K20, and K91) (Musselman et al., 2012). Although acetylation was discovered about 60 years ago, the first reader of acetylated lysine, a bromodomain, was discovered only in 1999 (Dhalluin et al., 1999). Till now, three types of protein domains able to “read” acetyllysine marks have been identified: bromodomains, DPF domain, and YEATS domain (Figure 1). Here, we will discuss these newly identified reader proteins of acetylation mark.

### 3.1 Recognition of H3K14Ac by the Bromodomain Module of RSC Chromatin Remodeler

Bromodomains are evolutionarily conserved domains that act as histone lysine acetylation readers. In humans, 61 bromodomains in 46 different proteins have been identified, and these proteins are part of transcription-regulating complexes, chromatin remodelers, and PTM writers. Based on the structure and sequence, bromodomains are divided into eight subfamilies (I–VIII) (Filippakopoulos et al., 2012). Even with little sequence homology, all bromodomains have a conserved structural fold consisting of four α-helices (αZ, αA, αB, and αC) (Figure 1A). The two highly variable loops, ZA and BC, joining these helices, form a deep hydrophobic acetyllysine binding pocket (Sanchez and Zhou 2009).

One of the yeast chromatin remodelers, the RSC complex, consists of seven bromodomains. Acetylation of histone H3 lysine at the 14th position enhanced RSC binding to nucleosomes and augmented the RSC remodeling activity (Duan and Smerdon 2014; Lorch, Maier-Davis, and Kornberg 2018). Recently, Chen et al. (2020) showed that out of seven bromodomains present in RSC (one in the Sth1 subunit and two each in Rsc1, Rsc2, and Rsc4 subunits), the C-terminal bromodomain of Sth1 is the primary domain responsible for recognizing H3K14Ac. ITC experiments using H3K14Ac containing H3_6–21_ peptide revealed that Rsc1 and Rsc2 have no significant interaction, while Rsc4 (dissociation constant of 263 μM) has a 16-fold weaker interaction than Sth1 bromodomain (dissociation constant of 16 μM). Further ITC results with an array of histone peptides containing different acetylation sites demonstrated that the Sth1 bromodomain could also strongly bind to H3K20Ac with similar *K*_*D*_ as that of H3K14Ac. Sequence analysis of these peptides revealed a conserved feature in both H3K14Ac and H4K20Ac peptides: the following two residues after lysine are hydrophobic, and the third one is a conserved arginine (K(Ac)ΦΦR motif, where Φ represents any hydrophobic amino acid). Mutation at the +1 and +2 position with neutral or polar amino acid and mutation of +3 arginine abolish the interaction between the peptide and Sth1 bromodomain.

**FIGURE 2:**
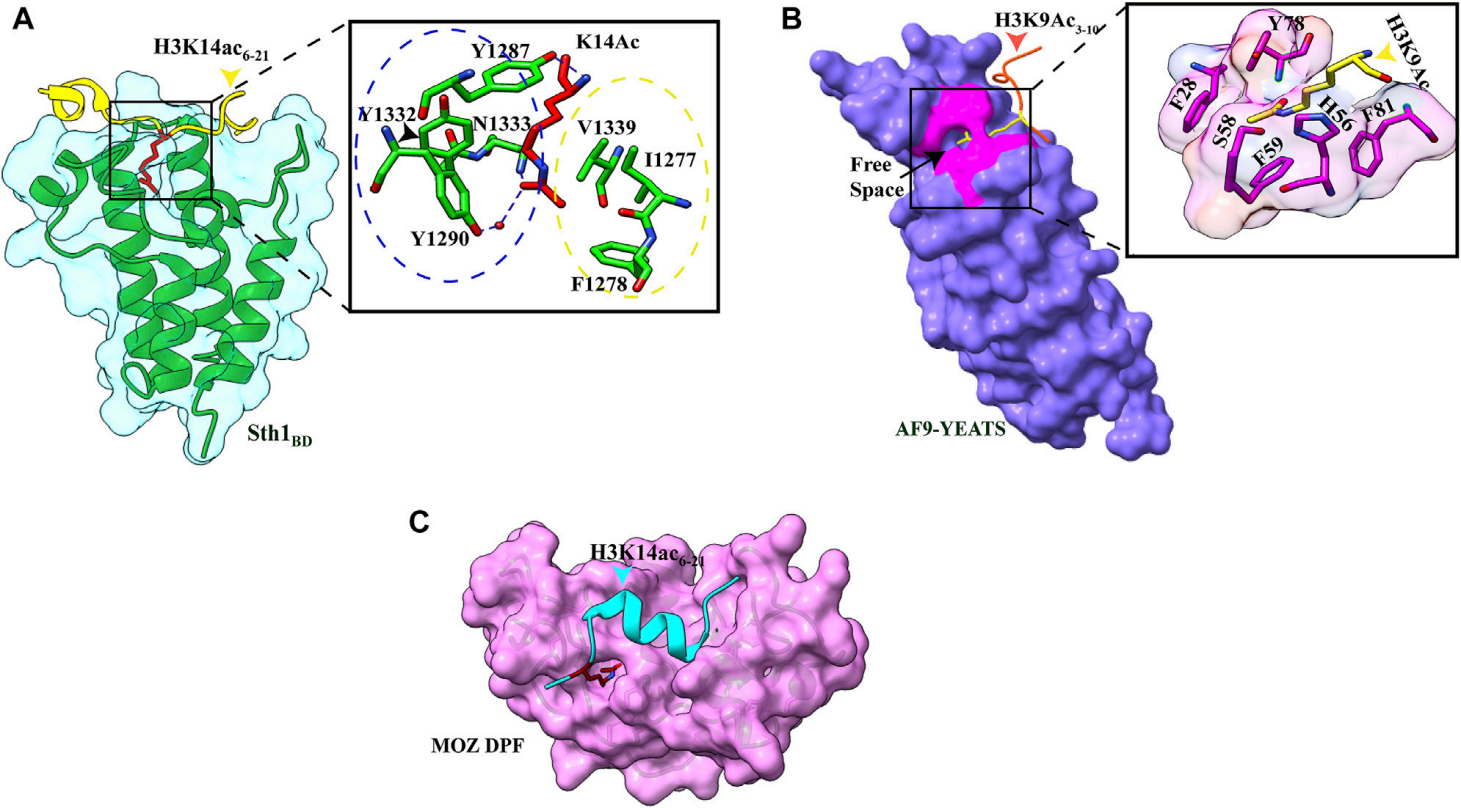
Readout of acetyllysine by different readers. **(A)** Left: overall structure of Sth1_BD_ (green ribbons) with H3K14ac_6–21_ (yellow color, K14 shown as red sticks). Right: close-up view of H3K14ac-binding sites of Sth1. H3K14Ac is shown in red color, and Sth1BD residues are shown in green color. Residues in the blue circle interact with the aliphatic side chain of K14, while residues in the yellow circle interact with the methyl group of acetyl mark. Hydrogen bonds are shown as blue dashed line (PDB ID: 6KMJ). **(B)** Left: overall structure of the AF9 YEATS domain (purple color) with H3K9Ac_3–10_ (orange–red color, K9 shown in yellow color). K9ac (yellow color) can be seen inserted into the narrow end-open pocket. Right: close-up view of interacting residues of H3K9Ac (yellow color) and AF9 residues (pink color). A serine (S58)-lined aromatic cage (F28, H56, F59, Y78, and F81) is formed in which the acetylated lysine snugly fits (PDB ID: 4TMP). **(C)**. Overall structure of H3K14Ac_3–15_ (stick model in cyan color) with MOZDPF (pink surface). K14ac (red color) can be seen inserted in the “dead end” pocket of MOZ protein (PDB ID: 4LLB).

Like other bromodomain-containing proteins, Sth1 has a hydrophobic pocket formed by four amphipathic α-helices in which K14Ac is inserted. In addition to hydrophobic contacts, interactions between the aliphatic side chain of K14 with three aromatic amino acids (Y1287, Y1332, and Y1290), the methyl group of acetyl mark with V1339, I1277, and F1278, and the hydrogen bond between the carbonyl oxygen of the acetyl group and N1333 of Sth1 are responsible for strong affinity (Figure 2A). The other three residues of the K(Ac)ΦΦR motif also make extensive hydrogen bonds and hydrophobic contacts with the Sth1 bromodomain, explaining the specific recognition of this motif by Sth1_BD._ Critical residues of hydrophobic pockets (Y1332 and N1333) are not present in Rsc1 and Rsc2 subunit bromodomains which explains their insignificant interaction with the H3K14Ac peptide (Chen et al., 2020).

More recently, the *C. elegans* homolog of Sth1, SMARCA4, was also shown to be highly selective for H3K14Ac and showed a similar binding affinity. The hydrogen-bonding and hydrophobic interactions are very well conserved in *C. elegans* SMARCA4 bromodomain and H3_7–20_K14Ac-modified peptide complex. In this case also, the K(Ac)ΦΦR motif is involved in the extensive electrostatic, hydrophobic, and hydrogen-bonding interactions that ensure specific and robust binding between SMARCA4 and H3K14Ac-containing peptides (Enríquez et al., 2021).

The RSC complex is the most abundant and well-characterized chromatin remodeler of the SWI/SNF family, comprising about 17 subunits in *Saccharomyces cerevisiae*. Like other remodelers, using its main catalytic subunit Sth1, the RSC complex catalyzes the ATP hydrolysis reaction and uses this energy to evict or side the histone octamer to expose DNA-binding sites on chromatin. At the H3K14Ac-enriched transcription start sites (TSSs), the RSC complex is recruited, which generates a nucleosome-free region enabling RNA Pol II to initiate transcription (Carey, Li, and Workman 2006; Lorch et al., 2011). Also, H3K14Ac is found at UV-irradiated DNA sites, which recruit the RSC complex to facilitate DNA repair by chromatin remodeling (Yu et al., 2005; Duan and Smerdon 2014).

### 3.2 Recognition of H3K9ac by the YEATS Domain

A study published in 2014 showed YEATS domain as a novel reader of histone acetylation marks. It is an evolutionarily conserved protein module from yeast to humans and is named after its founding domain-containing proteins, Yaf9, ENL, AF9, Taf14, and Sas5 (Masson et al., 2003). Three YEATS domain-containing proteins in *S. cerevisiae* and four proteins in humans are associated with transcription-regulating complexes, chromatin-remodeling complexes, and HAT complexes (Schulze et al., 2009). ITC and pull-down assay revealed that binding of the YEATS domain of AF9 protein to histone H3 tail is acetylation dependent, and the AF9 YEATS domain binds strongly to H3K9Ac (*K*_D_ of 3.7 μM) as well as to H3K27Ac (*K*_D_ of 7.0 μM) and H3K18Ac (*K*_D_ of 11.0 μM), however, to a lesser extent (Li et al., 2014). The crystal structure of YEATS domain with different acetylated histone peptides uncovered a unique serine-lined aromatic sandwich pocket for specific acetyllysine readout. The AF9 YEATS domain adopts an immunoglobin fold in which eight antiparallel β strands form a two-layer β sandwich, and H3K9Ac long side chain is inserted into a serine-lined aromatic cage formed in the cleft of loops L4 and L6 (Figures 1B, 2B). In the AF9 YEATS–H3K9Ac complex, the YEATS domain uses strands β2 and β7 and loops L1, L4, L6, and L8 to form extensive hydrogen bonds and hydrophobic interactions with the T3-S10 segment of H3. In addition to hydrogen and hydrophobic interactions, multiple aromatic residues in the acetyllysine binding pocket are involved in multiple sets of CH–π interactions, which collectively contribute to the stable binding (Figure 2B). Key residues involved in the generation of the aromatic cage are highly conserved among different YEATS domain-containing proteins from yeast to humans. The interaction of the YEATS domain and H3 tail is also highly dependent on amino acids flanking the K9, especially arginine, at the eighth position, as mutation at this site resulted in a 200-fold binding decline (Li et al., 2014).

AF9 is subunit of a large protein complex, Super Elongation Complex (SEC), which has been shown to mediate enhanced transcription of several loci in MLL-rearranged leukemias and developmental genes by releasing paused Pol II (Smith, Lin, and Shilatifard 2011). ChIP-seq and CoIP experiments suggested that the YEATS domain (N-terminal part of AF9) is critical for the recruitment of AF9 at the H3K9ac mark around the transcription start sites and C-terminal of AF9 is required for the interaction with other proteins of SEC complex *in vivo*. One of the critical interacting partners of AF9 is DOT1L, an H3K79 methyltransferase, and H3K79me3 mark is associated with active transcription. Several *in vivo* experiments revealed that AF9 is required for DOT1L recruitment at targeted genes and subsequent deposition of H3K79me3 to promote active transcription (Li et al., 2014).

### 3.3 Recognition of H3K14ac by the DPF Domain

The double PHD finger(DPF) domain, a subgroup of PHD (plant homeodomain) fingers, is a tandem of PHD fingers with a face-to-back orientation where two domains form a single structure. This domain has been found in two protein families, histone acetyltransferase MYST family proteins (MOZ or KAT6A and MORF or KAT6B) and subfamilies of SWI/SNF chromatin remodeler (BAF and PBAF complex). The DPF domain from all these proteins is homologous, and all the key residues are conserved, forming a highly similar secondary structure consisting of two antiparallel β-sheets followed by a C-terminal α-helix which is coordinated by two zinc atoms *via* Cys4-His-Cys3 motif in a cross-brace topology (Figure 1C). Two PHD fingers are linked with one another in a face-to-back orientation mediated by the interaction between glutamic acid and arginine in the α-helix of the first PHD finger. Although the PHD finger was originally recognized as a methylation mark reader, the DPF domain of DPF3b was shown to bind H3K14 acetylation mark. The structural aspects of acetyllysine–H3K14Ac interaction are discussed in the next section.

## 4 Acylations

**FIGURE 3:**
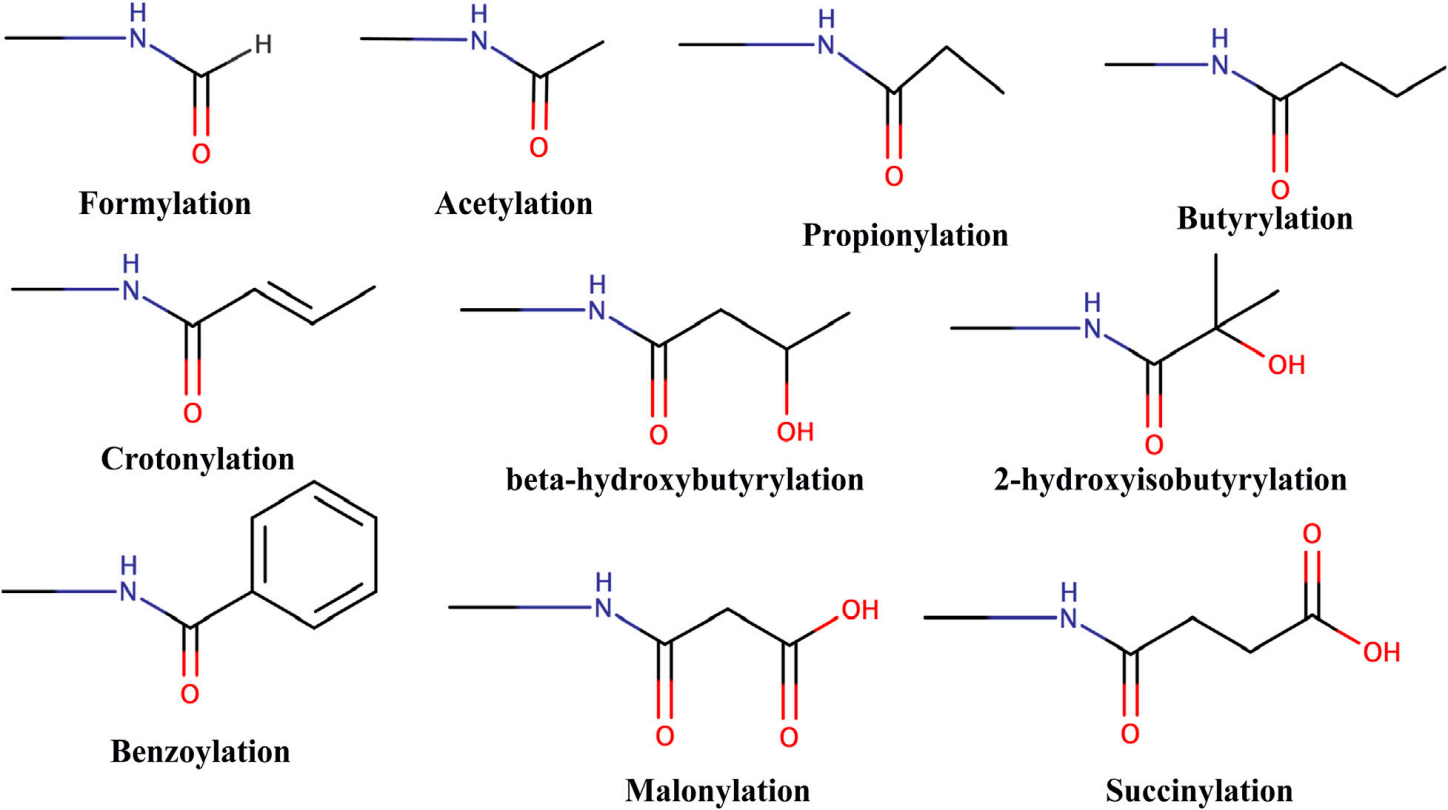
Chemical structure of different types of acylations.

The latest advancements in mass spectrometry revealed a wide array of acylation marks in histones apart from classical acetylation modification. These acyl marks include butyrylation (Kbu) (Chen et al., 2007), propionylation (Kpr) (Chen et al., 2007), crotonylation (Kcr) (Tan et al., 2011), succinylation (Ksucc) (Xie et al., 2012), malonylation (Kma) (Xie et al., 2012), 2-hydroxyisobutyrylation (Khib) (Dai et al., 2014), β-hydroxybutyrylation (Kbhb) (Xie et al., 2016), benzoylation (Kbz) (Huang et al., 2018), lactylation (Kla) (Zhang et al., 2019), glutarylation (Kglu) (Bao et al., 2019), and isobutyrylation (Kibu) (Zhu et al., 2021) (Figure 3). These modifications are derived from their respective acyl-CoAs, a product of different metabolic pathways. Therefore, these specific marks can identify the metabolic state of the cell and regulate chromatin dynamics and gene expression according to the need of the cell (Nitsch, Shahidian, and Schneider 2021). Also, various new studies suggest that these different acylation marks are important for eliciting specific epigenetic responses (Dutta, Abmayr, and Workman 2016).

Till date, no specific or selective writer, reader, or eraser for non-acetyl acylation modification has been identified so far. A recent study with high-throughput profiling of an acyl-CoA/protein using CoA/AcetylTraNsferase Interaction Profiling (CANTIP) revealed only known acetyl mark-interacting proteins (Levy et al., 2020). In fact, p300 lysine acetyltransferase (also known as KAT3B) has been shown to be able to catalyze the transfer of all types of different acyl groups (Nitsch, Shahidian, and Schneider 2021).

### 4.1 Recognition of Acyl Marks by Bromodomains

Given that bromodomain is a major protein module that reads lysine acetylation, an initial study found out that the bromodomain of bromodomain-containing protein (BRD4) was able to bind Kbu and Kpr but with very less affinity than Kac (Vollmuth and Geyer 2010). A more comprehensive study where 49 bromodomains were assayed for their binding affinity to different acyl-modified H3 peptides revealed that only bromodomains having larger binding pockets such as CECR2 and BRD9 were able to bind long-chain Kbu modification, and the second bromodomain of TAF1 was able to interact with Kcr, albeit with reduced affinity compared with Kac (Flynn et al., 2015). All these studies implied that bromodomains could read a few acyl marks, but these interactions are not strong and significant compared to acetyl modification.

### 4.2 Recognition of H3K9acyl/H3K18acyl/H3K27cr by the YEATS Domain

**FIGURE 4:**
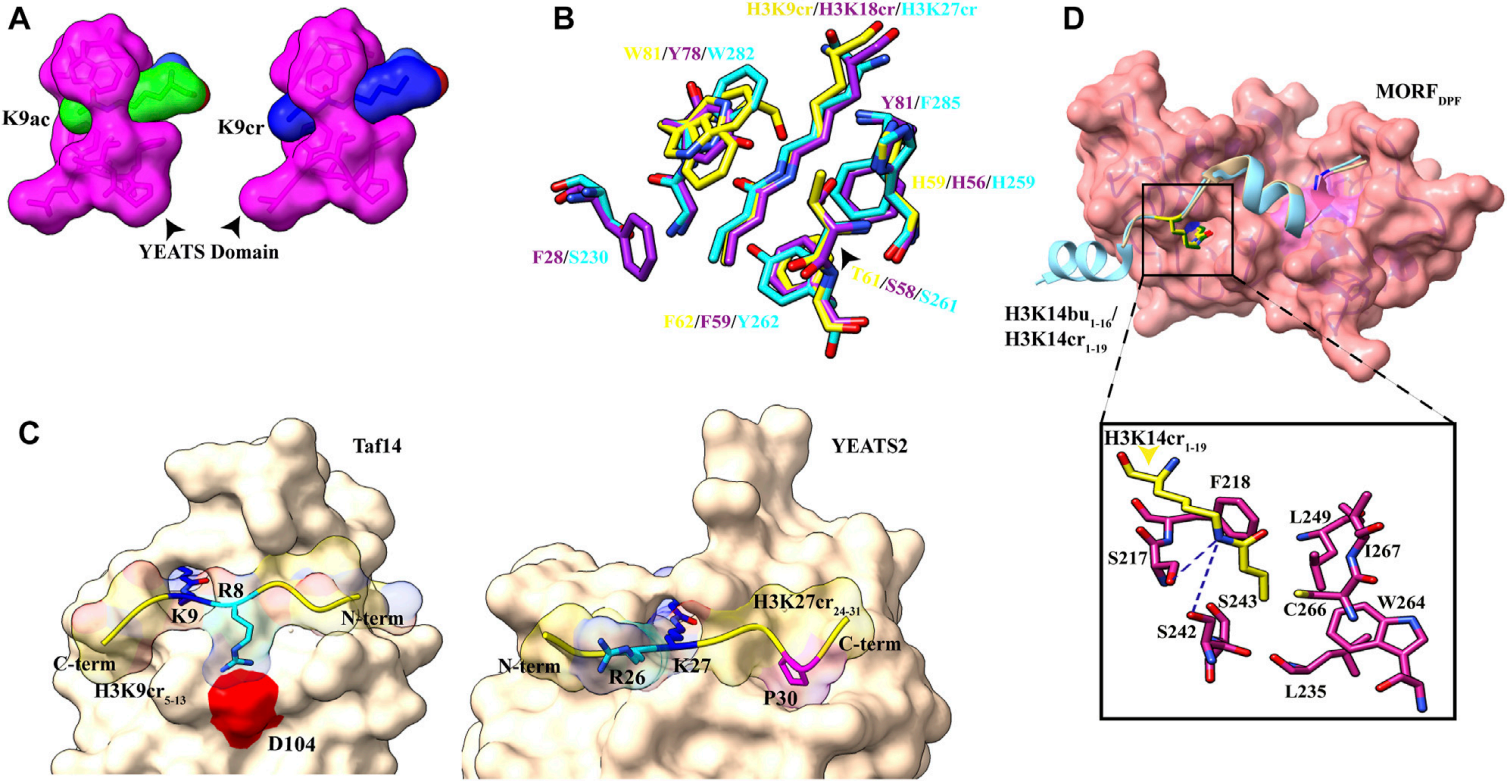
Readout of acyl marks by YEATS and DPF domains. **(A)** Longer chain of the crotonyl group (blue) can be accommodated in the Taf14 YEATS domain (magenta). (PDB ID: 5D7E and 5IOK). **(B)** Superimposition of residues involved in interaction among YEATS domains of different proteins: YEATS2 YEATS domain–H3K27cr (cyan; PDB ID: 5IQL), Taf14 YEATS domain–H3K9cr (yellow; 5IOK), and AF9 YEATS domain–H3K18cr (purple; 5HJD). **(C)** Opposite orientation of H3 peptides across Taf14 (PDB ID: 5IOK) and YEATS2 (PDB ID: 5IQL) proteins. In the Taf14–H3K9cr_5–13_complex, R8 (cyan) interacts with aspartate at 104th position. In YEATS2–H3K27cr_24–31_ complex, R26 (cyan) is facing away from the YEATS domain but proline at 30th position (magenta) makes contacts with the hydrophobic pocket. **(D)** Top: overall structure of the DPF domain of MORF protein with H3K14bu_1–16_ (wheat) and H3K14cr_1–19_ (cyan). Bottom: close-up view of amino acids involved in interaction between crotonylated lysine (yellow) and MORF_DPF_ protein (magenta) (PDB ID: 6OIE and 5B76).

In the crystal structure of the AF9 YEATS domain–H3K9Ac complex, a clear open space at the end of the aromatic sandwich cage led to the hypothesis that this open space can accommodate a large chain of bulkier acyl marks (Li et al., 2014) (Figure 1B). Further calorimetric titrations and NMR 2D ^15^N-^1^H heteronuclear single quantum coherence (HSQC) spectra revealed that, indeed, the AF9 YEATS domain could bind to the H3 tail peptides, which has crotonylation (cr), propionylation (pr), butyrylation (bu), and formylation (fo) modifications at K9, K18, and K27 positions with no significant binding for the H3K14 site (Li et al., 2016; Zhang et al., 2016). An increase in the hydrocarbon chain beyond the acetyl group resulted in 2.4-, 1.9-, and 1.4-fold binding enhancement for Kcr-, Kpr-, and Kbu-modified peptides, respectively. Similar studies with Taf14 and YEATS2 (subunit of ATAC histone acetyltransferase complex) established that the YEATS domain is a Kac reader module with the highest affinity to crotonylation mark (Andrews et al., 2016; D.; Zhao et al., 2016). The crystal structure of all three proteins in complex with H3 tail peptide having crotonylation mark showed a highly similar structure where the extended side chain of Kcr fits comfortably into the narrow end-open pocket of the YEATS domain (Figure 4A).

Two conserved aromatic residues in different YEATS domains (F62 and W81 for Taf14, F59 and Y78 for AF9, and Y268 and W282 for YEATS2) make a sandwich arrangement with the planar crotonylamide group, which crosses the β-sandwich cage at a 90° angle in a corkscrew-like manner (Figure 4B). This arrangement favors a novel “aromatic–π–aromatic” stacking (also called “π–π–π” stacking) (Klein et al., 2018). Additionally, extensive hydrophobic interactions, the amide−π interactions, the CH–π interactions, and electrostatic interactions (mainly of Cα and Cβ of the alkene moiety with the carbonyl oxygen of Q79) between the side chain of crotonylated lysine and pocket residues significantly contribute to specific recognition of Kcr by the YEATS domain (Krone et al., 2020).

Flanking residues in all three H3K9, H3K18, and H3K27 are conserved, sharing a common motif “A_(−2)_R_(−1)_KS_(+1)_.” An acidic aspartate residue of Taf14 and AF9 YEATS domains forms charge-stabilized hydrogen-bonding interaction with (*n*–1) arginine of H3K9cr peptide. Interestingly, in the YEATS domain of YEATS2 protein, this acidic aspartate residue is replaced by the neutral asparagine residue, which does not recognize “R_(−1)_.” While Taf14 and AF9 YEATS domain prefer H3K9cr and H3 N-terminal residues “K4-Q5-T6-A7-R8” have extensive interactions with loops L6 and L8 surface residues, YEATS2 binds H3K27cr more strongly and oppositely oriented C-terminal residues “S28-A29-P30-A31” fits nicely on the surface of L6 and L8 loops (Figure 4C). In the crystal structure of YEATS2, the YEATS domain in complex with H3K27cr revealed a hydrophobic pocket in YEATS2 in which H3P30 fits snugly and facilitates the correct positioning of H3K27cr, explaining the site specificity of YEATS2 (D. Zhao et al., 2016).

A recent study showed the role of histone crotonylation and Taf14 in the yeast metabolic cycle. In the yeast metabolic cycle, acetylation increases in high oxygen consumption state, followed by generation of crotonylation intermediates (Gowans et al., 2019). As the cells shift to a low oxygen consumption state, acetylation mark is replaced by crotonylation marks and in this LOC state H3K9cr and Taf14 repress the pro-growth genes, contrary to earlier studies showing their role in gene expression (Sabari et al., 2015).

### 4.3 Recognition of H3K14acyl by the DPF Domain

Pull-down assays and ITC experiments using an array of H3K14 bearing different acylations revealed that the DPF domain of DPF2 and MOZ HAT displays more affinity for H3K14cr, H3K14bu, and H3K14pr than H3K14Ac with crotonylation being the most favored (Xiong et al., 2016). On a similar line, a combination of fluorescence spectroscopy, NMR, and histone peptide pull-down assay established the specificity of the DPF domain of MORF HAT for H3K14cr and H3K14bu (Klein et al., 2017). In all the crystal structures solved for the DPF domain in complex with H3K14cr or H3K14bu, H3 tail peptide has extensive contacts with double PHD finger of the DPF module with segments H3_4–11_ and H3_17–25_ (in case of full length taken) adopting α-helical conformation (Xiong et al., 2016; Klein et al., 2017, 2019). The overall structural analysis revealed binding of the DPF module to acyllysine in a ping-pong-like manner with three characteristic interactions (Klein et al., 2019).

The first PHD domain of the DPF module forms a unique zinc-finger domain in which a hydrophobic pocket is formed at the β-sheet-2 surface. In the MORF (and MOZ) proteins, the hydrophobic pocket is formed by the amino acid residues I228–C230 of β-1, N235–G237 of β-2, and amino acid residues involved in zinc ion coordination S210 (S217), F211 (F218), L242 (L249), W257 (W264), C259 (C266), I260 (I267), and E261 (Figure 4D). The planar crotonylation group of H3K14 is inserted snugly into this hydrophobic reader pocket and stably positioned with the help of four water-mediated hydrogen-bonding interactions and four pairs of hydrophobic contacts. The structural and sequence alignment analysis of DPF domains showed that glycine residue G237 of β-strand-2 is a critical component of the hydrophobic pocket due to its free side chain. In classical PHD fingers, this glycine residue is replaced by bulky amino acids like phenylalanine or tyrosine, which fill the pocket and block large chain acylation mark insertion. One of the phenylalanines (F211 in MOZ and F218 in MORF) is in close proximity to the inserted crotonyl group and forms a π−π interaction between the C=C double bond of the crotonyl group and the aromatic ring of phenylalanine. This additional interaction is responsible for selectivity and strengthens the interaction between DPF domain and H3K14cr (Klein et al., 2019). Additionally, H3 residues R2 and K4 are inserted into two “acidic” pockets formed at the surface of β-1 sheet of the second PHD domain and held by hydrogen bonding and electrostatic interactions.

## 5 Serotonylation

Serotonylation is the attachment of the serotonin molecule to the glutamine residue of proteins. Serotonin [or 5-HT (hydroxy tryptamine)] is a monoamine with an abundant presence and diverse functions varying from neurotransmitter to hormone release and gastrointestinal motility. Additionally, serotonin has been shown to have the ability to covalently modify several proteins, including RacI, small guanosine triphosphatase, and fibronectin and thereby regulate their functions (Walther et al., 2003; Watts, Priestley, and Thompson 2009). The tissue transglutaminase 2 (TGM2) enzyme is responsible for conjugating serotonin to cytosolic proteins *via* the transamidation reaction (Hummerich et al., 2012).

It was previously known that TGMs could modify histones *in vitro* and do so very fast compared to some of the known native substrates (Abad, and Franco 1996). But recently, using the bio-orthogonal metabolic-labeling approach, Farrelly et al. (2019) showed that TGM2 can catalyze serotonylation of glutamine at the fifth position of histone H3 trimethylated lysine 4 (H3K4me3)-marked nucleosomes, resulting in the presence of combinatorial H3K4me3Q5ser *in vivo*.

### 5.1 Recognition of H3Q5ser by WDR5

**FIGURE 5:**
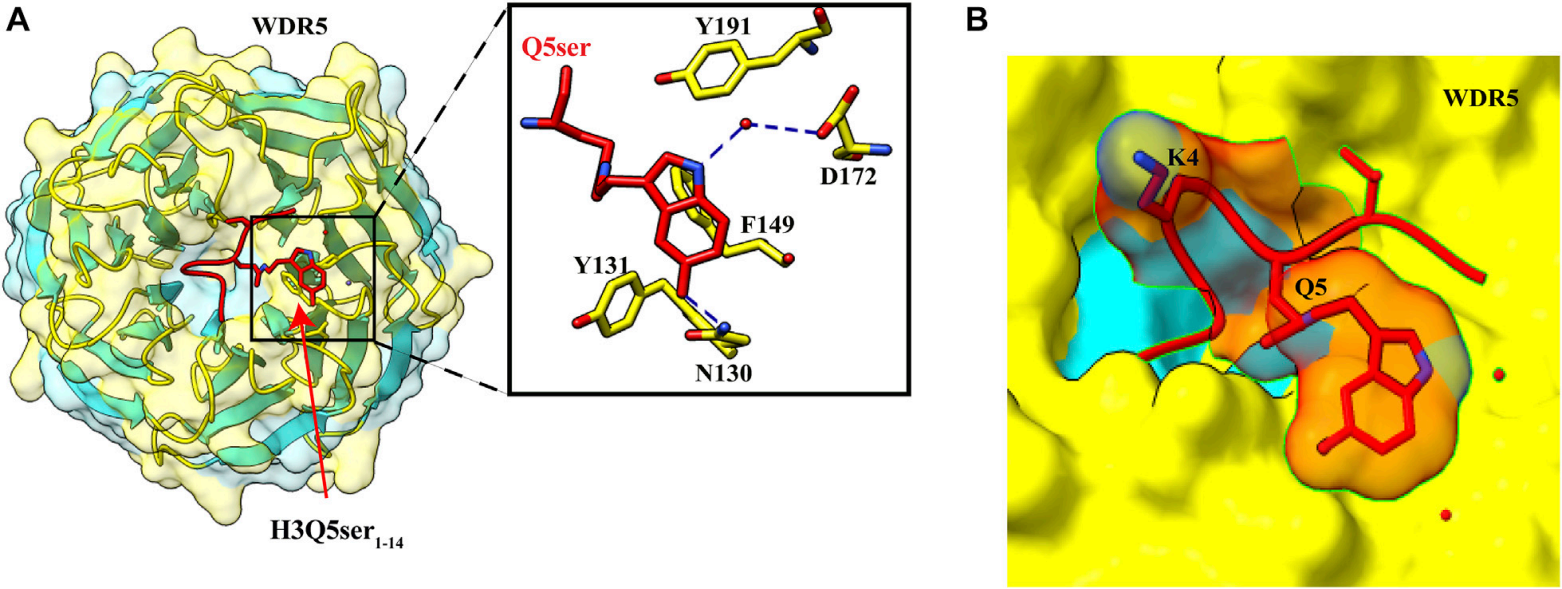
Recognition of H3Q5ser by WDR5. **(A) **Left: overall structure of WDR5_22–334_ in complex with H3Q5ser_1–14_ peptide (red). Right: enlarged view showing residues of WDR5_22–334_ (yellow) interacting with Q5ser (red). Hydrogen bonds are shown with blue dashed lines. **(B)** Lysine at fourth position is protruding away from the WDR5, resulting in no effect of trimethylation of K4 on the Q5ser–WDR5 interaction (PDB ID: 7CFQ).

In a pull-down assay, WDR5 was captured using H3 peptide with H3K4me3Q5ser dual marks as the bait, suggesting that WDR5 could be a potential reader of this modification (Farrelly et al., 2019). WDR5 is a core subunit of a histone methyltransferase enzyme; the MLL (mixed-lineage leukemia) complex is responsible for trimethylation of H3K4. Further pull-down assays and ITC experiment established that serotonylated H3Q5 enhances the binding of WDR5 by at least two-fold than that of unmodified H3, and H3K4 trimethylation mark has no significant effect on this binding, also supported by the observation that there was no electron density for the trimethyl group of K4 in the crystal structure of WDR5–H3K4me3Q5ser complex (Jie et al., 2022) (Figure 5B).

The crystal structure of WDR5–H3Q5ser complex revealed that the serotonyl group is placed in a shallow hydrophobic surface pocket of WDR5. The WDR5–Q5ser interaction is stabilized *via* a network of hydrogen bonds (one between OH group of serotonin with amide group N130 residue of WDR5 and another between amide group of serotonin with WDR5 D172 side chain) and van der Waals contacts (between hydrophobic moiety of serotonyl group and aromatic side chains of Y131, F149, and Y191 of WDR5) (Figure 5A). Additionally, R2 of H3 peptide also participates in this complex formation as it is anchored into a negatively charged central channel and interacts with WDR5’s F133 and F263 through cation–π interactions (Jie et al., 2022).

In neuroblastoma cells, upon recognition of H3Q5ser modification, WDR5 is recruited to promoter regions of oncogenic genes *GPX1*, *C-MYC*, and *PDCD6* that can promote tumor formation (Jie et al., 2022). Knockdown studies implied that serotonylation of H3Q5 is not dependent on H3K4me3; instead, there was a decrease in the level of H3K4me3 upon WDR5 knockout, also seen in the case of TGM2 knockdown, which may be due to less recruitment of MLL1 complex. H3K4me3Q5ser displays a ubiquitous pattern of tissue expression in mammals, with enrichment observed in the brain and gut, two organ systems responsible for the bulk of 5-HT production. Genome-wide analyses of human serotonergic neurons, developing mouse brain, and cultured serotonergic cells indicate that H3K4me3Q5ser nucleosomes are enriched in euchromatin, are sensitive to cellular differentiation, and correlate with permissive gene expression—phenomena that are linked to the enhanced function of TFIID (Farrelly et al., 2019).

## 6 Methylation

Histone methylation and its importance in transcription were first observed in the 1960s. There are three lysine methylation states: -mono, -di, and -tri (me1, me2, and me3); since methylation does not change histone’s charge configuration, the primary function of these methylations is to interact with effector molecules that specifically recognize these modifications. Generally, all other histone modifications are specific for the active or repressed state, while in methylated chromatin it depends on its methylation state and the modification position. For example, H3K4, H3K36, and H3K79 methylations are considered to mark active transcription (Heintzman et al., 2007), whereas H3K9, H3K27, and H4K20 methylations are associated with silenced chromatin states (Bernstein et al., 2005; Barski et al., 2007).

**FIGURE 6:**
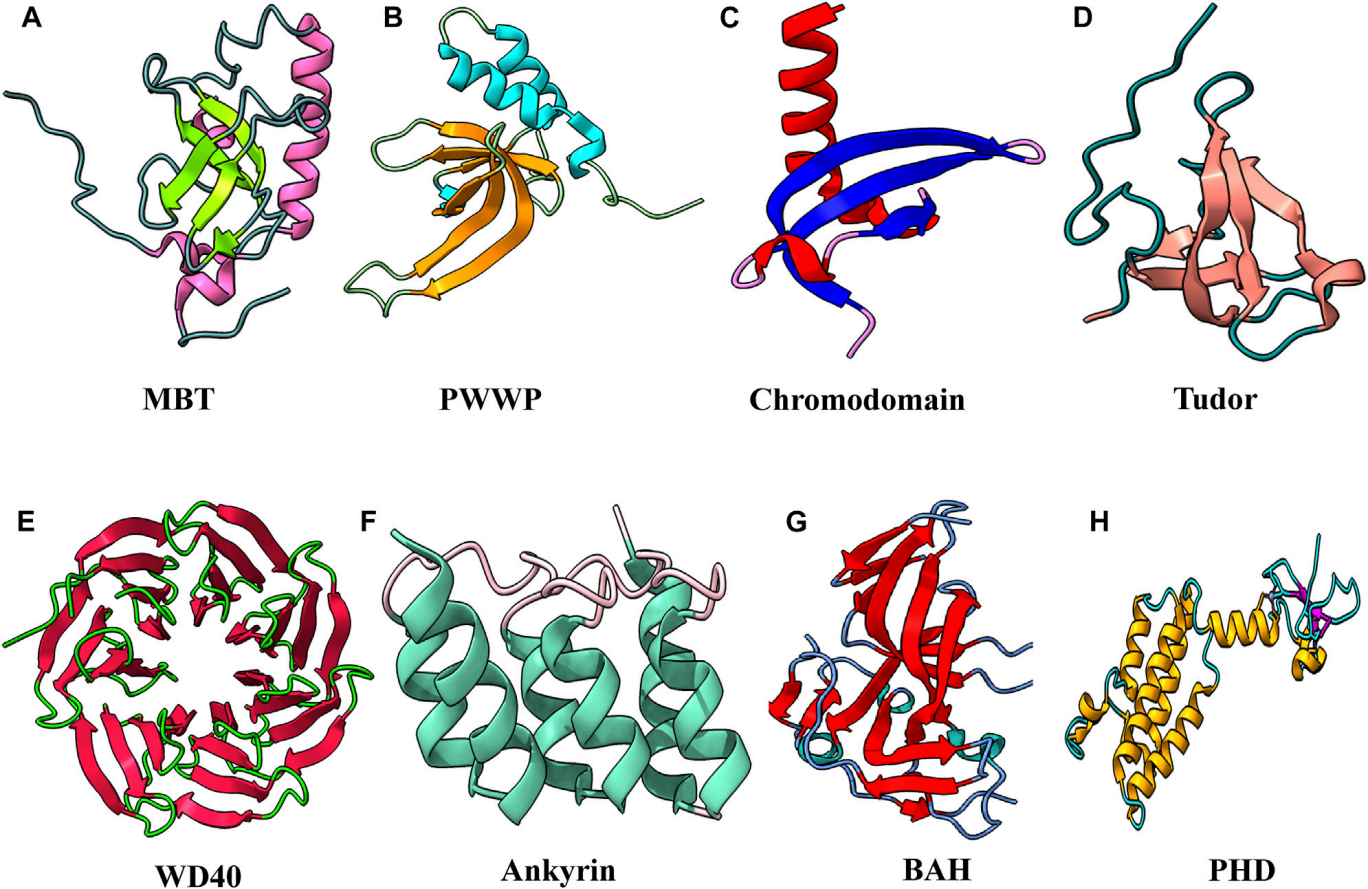
Structural features of domains capable to “read” methyl marks. **(A)** Second MBT domain of human KIAA1617: β-strands in light green and α-helix in pink (PDB ID: 1WJR). **(B)** PWWP domain of Pdp1 (PDB ID: 2L89). **(C)** Chromodomain of MPP8 (PDB ID: 3QO2). **(D)** Tudor domain of human PHF20 (PDB ID: 3SD4). **(E)** WD40 repeats of MYC (PDB ID: 6U8O). **(F)** Ankyrin repeats of human liver-type glutaminase (PDB ID: 5U0K). **(G)** Bromo-adjacent homology domain of human polybromo-1(PDB ID: 6OXB). **(H)** PHD of human BPTF protein (PDB ID: 2F6N).

Methyllysine-specific readers have a peculiar characteristic: they recognize histone modification by an aromatic cage that comprises two to four aromatic amino acids. These aromatic amino acids in some complexes are perpendicular to each other, which helps encircle the entire lysine methylation. The compartment of the aromatic cage defines whether mono-, di-, or tri-methylation state interacts with it. Therefore a small compartment limits its interaction with a higher methylation state because of steric hindrance, while a large compartment favors the interaction with a higher methylation state. Interaction in the compartment between the methylammonium group and the aromatic cage is stabilized by cation–π interactions and the hydrophobic and van der Waal interactions. Amino acids surrounding methyllysine play a vital role in the reader’s specificity for a particular methylated lysine. Some readers show very low specificity, while others are specific for a specific methylated state. Beyond caging of the methyllysine, the mechanism of recognition of surrounding residues varies among readers. A number of evolutionarily conserved domains were discovered that interact specifically with the methylated histone. These “reader” proteins contain methyl-lysine-binding motifs, including PHD, chromo, Tudor, PWWP, WD40, BAH, ADD, ankyrin repeat, and MBT domains (Figure 6). These readers can distinguish target methyllysine based on their methylation state and surrounding amino acid sequence (Musselman et al., 2012).

### 6.1 Royal Superfamily

In this superfamily, domains are structurally related and have β-barrel topology. It is believed that they come from a common ancestor, which has the conserved binding ability with the methylated substrate. All the family members consist of a slightly curved β-barrel with three β-strands followed by a short 3_10_ helix, and different members are distinguished based on additional strands or helices. This family includes MBT, Tudor, chromodomain, and PWWP (Yap and Zhou 2010).

#### 6.1.1 Recognition of H4K20me1/me2 by the MBT Domain of L3MBTL1

Isothermal titration calorimetry (ITC) assay using different methylation states of H4K20 peptide revealed that the MBT domain of human L3MBTL1 displays more affinity toward mono- and dimethylation states and does not bind to unmodified and trimethylated histone peptide. However, the binding is relatively of low affinity (*K*_D_ = 5–40 μM) and promiscuous (Min et al., 2007). The crystal structure of L3MBT1 with three MBT domains bound to histone peptide of 11 residues (H4 residue 15–25 with H4K20me2) shows the similar structure of all MBT domains assembled in a triangular shape. The structure is consistent with previously known structures of the MBT domain. It consists of four β-strands that form β-barrel and other extended arms of helices and a shorter strand (Wang et al., 2003). L3MBTL1 has three repeats of the MBT domain; however, only the second MBT domain binds to methyllysine (Santiveri et al., 2008; Eryilmaz et al., 2009; Li et al., 2007; Min et al., 2007). Superimposition of all three MBT domains revealed that the shorter side chain of cysteine 363 in the aromatic cage of the second MBT was replaced by bulky amino acids in MBT1 and MBT3. Thus, steric hindrance prevents the binding of MBT1 and MBT3 with methylated lysine. It also preferentially reads mono- and dimethylation by the cavity insertion mode, and specificity toward a lower methylation state is because of the aspartate residue present in the aromatic cage. Typical interactions in the aromatic cage are cation–π between aromatic residues and positively charged methylammonium of methyllysine. Additionally, aspartate binds to methylammonium *via* hydrogen bonding in the MBT domain.

In contrast, the flanking residues of the peptide substrate show little interaction with the protein. Only two water-mediated hydrogen bonds are present, first between the backbone carbonyl group of H18 and Y386 of the protein and a second between the carbonyl NH group of K20 and N358 of the protein.

H4K20 methylation was previously linked to chromatin compaction. However, H4K20me1 was found in the actively transcribing genes, contradicting the previously suggested role of methylation in chromatin compaction. A recent study revealed that H4K20me1 nucleosomal arrays were less compacted than H4K20me0 and H4K20me3 nucleosomal arrays, and the H4 tail was more dynamic in K20 mono-methylation. This study suggests that mono-methylation of H4K20 facilitates the opening and accessibility of chromatin (Shoaib et al., 2021).

#### 6.1.2 PWWP Domain

PWWP domain was first identified in the WHSC1 protein that contains the 100–130 amino acid structural motif (Stec et al., 1998). It also has a conserved Pro-Trp-Trp-Pro motif, and it consists of five β-strand barrels packed against the helical bundle. Despite the sequence conservation in different proteins, some variation in the PWWP motif can occur. For example, methyltransferase DNMT3a/b has SWWP (Qiu et al., 2002), and hepatoma-derived growth factor (HDGF) has a PHWP motif instead of a PWWP motif (Sue et al., 2004). It was initially identified as the DNA-binding protein; however, its similarity to the Tudor and chromodomain suggests that it might have the ability to bind methylated lysine. DNMT3a protein responsible for DNA methylation contains the PWWP domain. This domain is also known to interact with methylated histone tails, which led to assumptions that it might have dual binding to histone tails and the dsDNA.

Pdp1 protein, which contains PWWP domains, binds to methylated lysine, and dsDNA was seen by fluorescence polarization assay (FPA) (Qiu et al., 2012). The binding studies showed that PWWP domains of Pdp1 bind to the H3K20 trimethylation. After that, many other PWWP domains were shown to exhibit the binding with methylated lysine. Except for Pdp1, all the proteins containing PWWP domains bind specifically to the H3K36 methylation, suggesting its role as the H3K36 methylation sensor (Vezzoli et al., 2010; van Nuland et al., 2013; Wang et al., 2020). As it binds to the trimethylation state of lysine, it suggests that the binding cavity of the PWWP domain is wider to accommodate the bulkier me3 group than the MBT domain, which can only interact with mono- and dimethylated states. Therefore, this domain shows less specificity for the degree of methylation state.

Structural analysis of Pdp1 revealed that the aromatic cage is formed by Y63, W66, and F94. Cation–π interactions are used by the Pdp1 PWWP domain to recognize the trimethylated lysine at 20th position, and two residues (D97 and N99) from the loop between β3 and β4 form an extensive network of hydrogen bonds with the histone H4 tail residues (R19, K20, and V21) (Qiu et al., 2012). Y63, W66, and F94 amino acid side chains are perpendicularly oriented, forming an aromatic cage accommodating the trimethylammonium group. Mutations of the residues that compose the aromatic cage abolish methylated histone peptide binding.

#### 6.1.3 Recognition of H3K9me3 Marks by HP1 Chromodomain

The chromatin organization modifier domain (chromodomain) is the smallest member of this superfamily. The structural motif is based on the HP1 fold, consisting of three curved antiparallel β-sheets followed by the α-helix (Ball et al., 1997). There are approximately 55 proteins identified which contain chromodomain. These chromodomain-containing proteins were associated with chromatin silencing. These proteins are divided into two groups: canonical (based on HP1 structure) group includes polycomb proteins Cbx1-9, CMT1-3, and CYD; and noncanonical, including CHD1-8, RBBP1, and HRP1.

HP1 and polycomb proteins recognize H3K9me3 (Jacobs and Khorasanizadeh 2002) and H3K27me3 (Min, Zhang, and Xu 2003), respectively, through their ARKS/T motif. The histone tail inserted between two strands forms the complete β-barrel in both proteins. This insertion of the histone tail is stabilized by the electrostatic interactions and the hydrogen bond between the backbone. This interaction involves seven amino acids preceding methyllysine and one following amino acid. This recognition method prefers the recognition of trimethylation over mono- and dimethylation. Recent cryo-EM structure of H3K9me3 di-nucleosome with HP1α, HP1β, and HP1γ revealed how heterochromatin is organized. In this structure, di-nucleosomes trimethylated at K9 are bridged by two symmetric molecules of HP1. Linker DNA between the nucleosome is not interacting with the HP1, which leaves linker DNA to interact with ACF (ATP-utilizing chromatin assembly and remodeling factor) (Machida et al., 2018).

The noncanonical chromodomain proteins are based on the chromo ATPase/helicase-DNA-binding (CHD) protein. They contain two chromodomains, both at the N-terminal, for example, SNF2-type helicase, which is involved in chromatin remodeling. CHD7 specifically recognizes H3K1me1 as the enhancer for the gene.

#### 6.1.4 Recognition of H3K4me3K9me3 Bivalent Mark by the Tudor Domain of Spindlin1

Tudor domains are structurally diverse and mediate protein–protein interactions. Tudor domains interact with all methylation states. This domain consists of approximately 60 amino acids of four or five β-strands which form a β-barrel structure followed by one or two helices (Selenko et al., 2001). Tudor domain-containing protein interacts with the H3K4me3 (Wang et al., 2011; Yang et al., 2012), H3K9me2 (Arita et al., 2012), H3K36me3 (Cai et al., 2013), and H4K20me3 (Hirano et al., 2012). Almost 30 known proteins have this Tudor domain, including JMJD2, 53BP1, SGF29, Spindlin1, UHRF1, PHF1, OHF19, LBR, and TDRD3 (which recognizes methylated arginine residues). Proteins in this family are involved in various biochemical processes like DNA methylation, nonhomologous end joining, DNA damage and repair, transcription activation and repression, and rRNA expression.

To meet the growing demand for ribosomes in rapidly growing cells, more copies of rRNA are produced at a greater transcription rate. The repressive histone methylation marks present on H3K9/K27 and H4K20 in the heterochromatin region are linked to rRNA transcription suppression. Because it has been established that H3K4 methylation is required for active gene expression, the cell must establish H3K4 methylation and H3K9 demethylation to convert the suppressed rRNA expression to active expression. Although H3K4 and H3K9 trimethylation are mutually exclusive, bivalent H3K4me3 and H3K9me3 have been documented in specific cell types (Mikkelsen et al., 2007; Bilodeau et al., 2009; Rugg-Gunn et al., 2010; Matsumura et al., 2015). It was previously reported that KDM4A and KDM4C recognize the H3K4me3 *via* their Tudor domain and help in the demethylation of H3K9me3. This study suggests that these methylation marks can coexist on the same H3 N-terminal tail and functionally crosstalk (Huang et al., 2006; Yamamoto, and Fujimori 2016). It was also previously known that euchromatin rRNA genes contain bivalent mark H3K4me3K9me3 (Murayama et al., 2008). So for rRNA synthesis, the bivalent mark of H3K4 and H3K9 trimethylation is needed.

**FIGURE 7:**
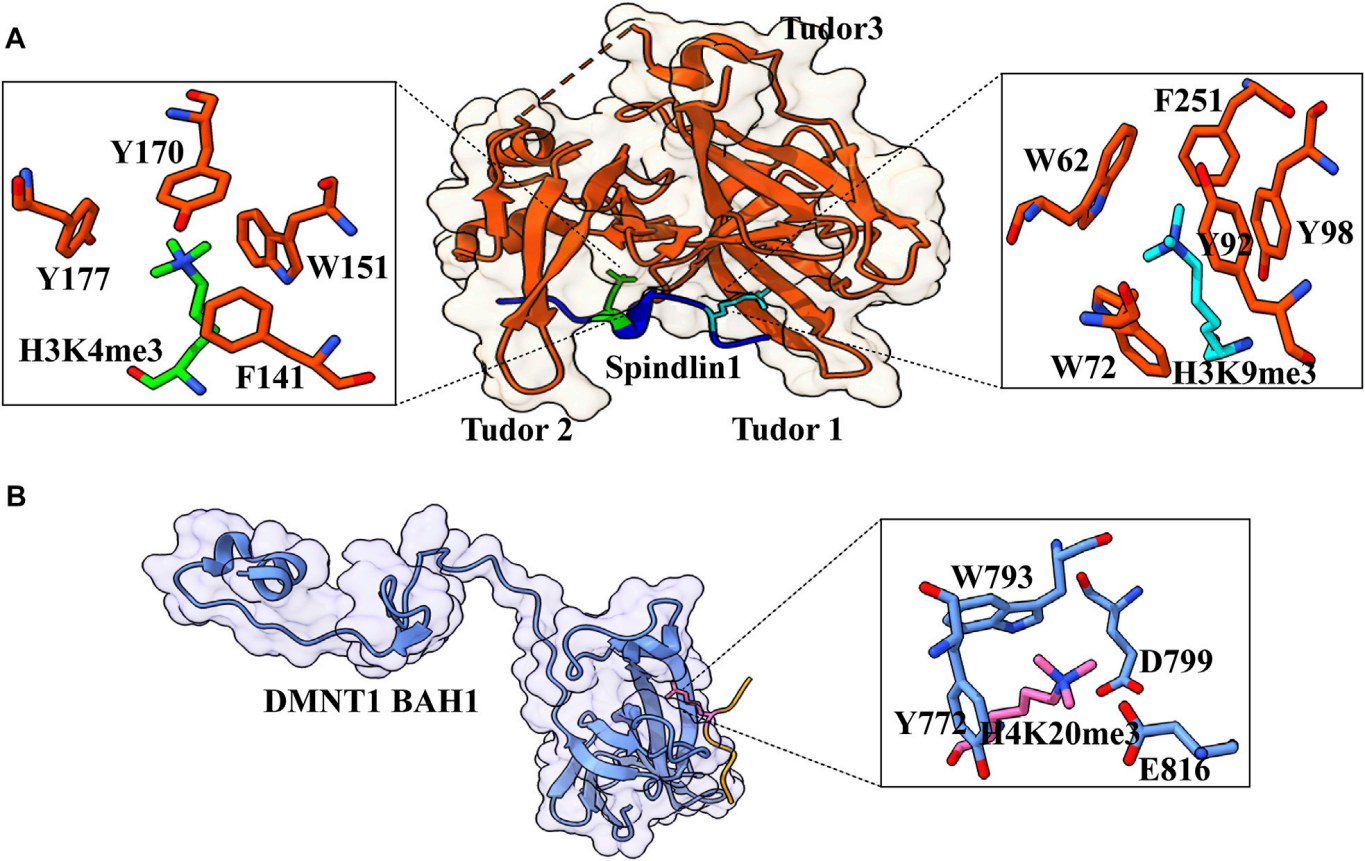
Interaction details of Spindlin1 and DNMT1 with trimethylated histone peptides. **(A)** Structure of Spindlin1 with H3K4me3K9me3 peptide; Spindlin1 shown in orange, H3K4me3 in green and H3K9me3 in cyan. Key residues involved in the interactions are depicted as a ball‑stick model shown in an enlarged view (PDB ID: 7CNA). **(B)** Structure of DNMT1 BAH1 with H4K20me3 peptide; DNMT1 BAH1 is shown in light blue and H4K20me3 in pink (PDB ID: 7LMK).

The Tudor domain is found in Spindlin1, a protein that aids in rRNA expression. Splindin1 creates a complex with C11orf84 that recognizes the bivalent mark on the histone H3 tail. Tudor 2 domain residues F141, W151, Y170, and Y177 form an aromatic pocket for trimethylated K4, whereas Tudor 1 domain residues W62, W72, Y91, and Y98, as well as Tudor 3 domain residue F251, recognize trimethylated K9 (Figure 7A) (Du et al., 2021). In addition to cation–π interactions formed by dual methylated lysine, the N-terminal amino group of H3A1 forms a hydrogen bond with the side-chain carboxylate group of D189, and guanidino moiety of H3R2 is ion-paired with the side-chain carboxylate group of D184 (Du et al., 2021). This bivalent recognition further helps in the dislocation of HP1 from the rRNA chromatin, which relaxes the chromatin and helps in the recruitment of RNA polymerase I, which leads to the expression of rRNA. Spindlin1 Tudor 3 domain is responsible for the binding to C11orf84, while the other two Tudor domains act in concert to recognize a noncanonical bivalent histone mark H3K4me3K9me3.

### 6.2 The WD40 Repeats

WD40 repeats are present in proteins showing very diverse protein–protein interactions. WD repeats usually have 40–60 amino acids and conserved tryptophan–aspartate (WD) residue. This motif can be found in each blade of a WDR domain. The structural plasticity of WDR domains allows them to keep their β-propeller fold even after deletion of WD repeats, which can range from five to eight but are generally seven (Böcskei, and Polgár 1998; Juhász et al., 2005). Each of these repeats folds into a four-stranded β-sheet, and these propellers are large, usually containing ∼300 amino acids.

SET1 methyltransferase (catalyze methylation of H3K4) subunit WDR5 contains seven WD repeats to form the β-propeller structure. SET1 needs WDR5 for its assembly and activity. First, it was found that WDR5 binds to H3K4me2 and me3. However, the crystal structure of WDR5 with the unmodified, mono-, di-, and trimethylated H3K4 reveals that WDR5 interacts with H3R2 and acts as an arginine reader. WRD5 interacts with lysine only through E332, present at the protein’s surface. WRD5 anchor as the arginine pocket binds to the unmodified and demethylated arginine residue (Dharmarajan et al., 2012). It was proposed to act as a histone modification intermediate that binds to arginine and presents lysine for methylation.

### 6.3 Recognition of H3K9me1/me2 by Ankyrin Repeat Domain of G9a

It is a ∼33 amino acid repeat, and each repeat consists of helix–loop–helix structure with a β-hairpin/loop region; and in most proteins, 4–7 repeats are present, which stack onto each other to form the right-handed solenoid-like structure that helps in the protein–protein interaction (Sedgwick and Smerdon 1999). A short-distance interaction between inter α-helices helps form the solenoid, forming a typical globular protein shape. The structure of the AR domain is stabilized through inter- and intra-helical hydrophobic and hydrogen bonding *via* polar residues at the N-terminal. Hydrophobic interactions between P5 and H7 form the L-shape of the domain, and hydrogen bond between T4 and H7 facilitates the formation of β-hairpin with the adjacent loop (Yuan et al., 2004).

The H3K9me1 and H3K9me2 peptides bind to G9a ankyrin repeats with low affinity (*K*_D_ = 14 and 6 mM, respectively), with a crystal structure of ankyrin repeats–H3K9me2 peptide complex was solved. The dimethylammonium group of K9me2 is placed into an aromatic pocket lined by three tryptophan residues and glutamate while the H3 peptide is sandwiched between β-turns and the fourth and fifth helices ankyrin repeats (Collins et al., 2008). Peptide residues 9–11, which comprise K9me2, are involved in intermolecular recognition. The formation of complexes is hampered by mutations of certain peptide or aromatic cage residues. G9a’s SET domain-mediated methyltransferase activity is unaffected by changes in methyllysine recognition by the ankyrin repeat, indicating that the reading and writing domains work independently.

### 6.4 The Bromo Adjacent Homology Domain

Bromo adjacent homology (BAH) domain-containing proteins are frequently associated with chromatin functions, and mutations in these domains can result in diseases. BAH has been found to have various roles in chromatin biology, including protein–protein interaction, identification of methylated lysine, and DNA methylation. They have six tandem repeats of the bromodomain at the N-terminus, followed by the repetitive sequence motif of an unknown function; therefore, these are referred to as bromo adjacent homology domains. The majority of BAH domain proteins that have been identified have specific chromatin links, such as nucleosome remodeling and histone and DNA modifications.

BAH has an essential role in DNA methylation, which is an epigenetic mark that catalyzes the addition of the methyl group to the fifth position of cytosine (5-methylcytosine) (Suzuki and Bird 2008). Each round of DNA replication produces hemimethylated DNA, which must be converted to fully methylated DNA before the next round of replication, or the methylation marks will be lost. The family of DNA methyltransferases (Dnmts) mediates the transfer of the methyl group from *S*-adenyl methionine to the fifth position of cytosine at the CpG dinucleotide (Moore, Le, and Fan 2013). DNMT1 has N-terminal regulatory and C-terminal catalytic domains. Human Dnmt1 (1,616 amino acids) has two regions: N-terminal regulatory domain (1–1,139) and C-terminal catalytic domain (1,140–1,616). It also has several regions in N-terminal like CXXC (Bestor 1992), replication focus targeting sequence (RFTS) (Leonhardt et al., 1992), and BAH domain (Callebaut, Courvalin, and Mornon 1999).

H4K20 trimethylation is a significant heterochromatin mark that suppresses repetitive sequences in the human genome. By recognizing H4K20me3 *via* its first Bromo adjacent homology domain, DNMT1 promotes DNA methylation. The structure of DNMT1 with H4K20me3_14–25_ peptide shows that the side chain is inserted into a pocket created by DNMT1 BAH1 Y772, W793, D799, and E816 (Figure 7B) (Ren et al., 2021). The side chains of DNMT1 BAH1 D765 and E818 are bidentate hydrogen-bonded to the backbone amides of H4. BAH1 binds to H4K20me3, generating a change in the structure of DNMT1 that allows the autoinhibition linker to be displaced. DNMT1 can break away from the linker’s autoinhibition and get activated. H4K20me3 mark is put just after the S-phase of the cell cycle; therefore, H4K20me3 patterns may differ in new histones and parental histone. For a fact, recycled histone H4K20 is extensively methylated throughout replication, but fresh histones are only methylated during the G2/M phase (Ren et al., 2021). In this context, only the parental histones probably facilitate the DNMT1 activation, which lasts beyond the S-phase. This mechanism occurs alongside the UHRF-1-specific S-phase maintenance of DNA methylation (Rothbart et al., 2012).

### 6.5 PHD Domain

The PHD finger is a short zinc-binding module with a high cysteine content but few secondary structural elements that are now being characterized as a protein–protein or protein–phospholipid interaction domain. The PHD finger is a 50–80 amino acid motif composed of a two-strand antiparallel β-sheet and α-helix linked to the Cys4-His-Cys3 motif in a cross-brace shape by two zinc atoms (Bottomley et al., 2005; Elkin et al., 2005). The structure and function studies on PHD domain-containing proteins like BPFT (Li et al., 2006), IGN2 (Peña et al., 2006), and YNG1 (Taverna et al., 2006), which bind to H3K4 higher methylation state, provide the sequence and methylation state-specific recognition mechanism. Because of their ability to read numerous different post-translational modifications simultaneously, PHD proteins are some of the best examples of the “reader” class of proteins in combinatorial control of transcription. PHD fingers are found on a number of proteins involved in chromatin remodeling. Due to their frequent occurrence near other known chromatin interaction domains (bromodomains, PWWP domains), it was thought that PHD fingers could recognize histone modifications. In the various structures of PHD fingers that have been solved, the H3K4me3 mark can be found on most peptides. The modified histone peptide forms a strand that integrates into the PHD finger’s existing antiparallel sheet, and the majority of the complex structures have a similar topology.

## 7 Conclusion

The advancement in mass spectrometry and structural biology field in the past few years has revolutionized the field of epigenetics, resulting in an ever-growing list of PTMs. At the same time, the list of proteins having the capability to write, read, and erase these epigenetic marks is also growing, further expanding the complexity of epigenetics. For example, the identification of several short-chain acylation marks and their readers are critical in linking the metabolic state of the cell to gene regulation as most of the substrates for these PTMs are derived from different metabolic reactions. In the past 20 years, a lot of efforts have been put into understanding the molecular and structural mechanism of PTM readouts by their cognate reader proteins. These studies have identified a wide range of interacting modes, including cation–π and π–π–π stacking interactions (Klein et al., 2018; Du et al., 2021). Selective recognition of PTM by its cognate reader or multivalent readout of multiple PTMs on the same or different tails by a group of readers targets the protein or protein complex at targeted genomic sites and performs the downstream function. A deep understanding of these PTMs and their recognition by reader proteins is critical as any misregulation of these recognition mechanisms can lead to several human disorders like cancer. In case of misregulation, in-depth characterization of these binding mechanisms can aid in developing specific epigenetic-driven therapeutic targets. From the structural point of view, we have a solid knowledge base of different PTM–reader interactions. However, there are some questions that remain to be explicitly answered, concerning selectivity and specificity, like how the same reader can differentiate between acetyl and acyl modifications *in vivo*. Although a large number of PTMs have been identified and characterized, their reader partner is still to be characterized. Maximum structural studies on reader–PTM interactions till date used only short peptides. Therefore, structural elucidation of reader–PTM interaction at the nucleosomal level and further at the nucleosomal array level remains a major challenge. With improving structural biology tools and computational methods in tandem, it will be easier to overcome the existing limitations and answer questions associated with the molecular mechanism of “decorated tails” recognition by the epigenetic machinery.
